# Impact of Injection Level on Analgesic Efficacy of Adductor Canal Block Following Total Knee Arthroplasty: A Prospective Observational Study

**DOI:** 10.3390/jcm15135167

**Published:** 2026-07-02

**Authors:** Talha Karatas, Fethi Akyol, Hakan Gokalp Tas, Tulay Ceren Olmezturk Karakurt, Seyma Selin Aydin, Onur Isik, Taha Emre Otugen, Ufuk Kuyrukluyildiz

**Affiliations:** 1 Department of Anesthesiology and Reanimation, Kovancilar State Hospital, Elazig 23850, Turkey; talha1905-74@hotmail.com; 2 Department of Anesthesiology and Reanimation, Faculty of Medicine, Erzincan Binali Yildirim University, Erzincan 24100, Turkey; fethi24@windowslive.com (F.A.); hakangokalptas@hotmail.com (H.G.T.); drcerenkarakurt@gmail.com (T.C.O.K.); drufuk2001@gmail.com (U.K.); 3 Department of Anesthesiology and Reanimation, Susehri State Hospital, Sivas 58600, Turkey; selin.tkrl@gmail.com; 4 Department of Anesthesiology and Reanimation, Duzce Cagsu Hospital, Duzce 81030, Turkey; dr.onurisik@gmail.com; 5 Department of Anesthesiology and Reanimation, Erzincan Mengucek Gazi Training and Research Hospital, Erzincan 24100, Turkey

**Keywords:** adductor canal block, total knee arthroplasty, post-operative pain, nerve block, analgesics, opioid

## Abstract

**Background/Objectives:** Following total knee arthroplasty (TKA), adductor canal block (ACB) is usually used for postoperative analgesia; however, the optimal injection level is yet unclear. This study tried to determine how the injection level of the ACB had an impact on postoperative analgesic efficacy and functional results after total knee arthroplasty. **Methods:** A total of 108 patients undergoing unilateral total knee arthroplasty under spinal anesthesia were included in this prospective observational study and were categorized according to the level of ultrasound-guided adductor canal block performed (proximal [Group I], mid [Group II], or distal [Group III]). The visual analog scale (VAS) was used to measure postoperative pain at 1, 4, 8, 12, and 24 h. Additionally, functional pain throughout 24-h standardized static and dynamic tests was assessed. We noted 24-h opioid consumption, motor strength levels, and sensory and motor block features. **Results:** During each time point, the distal group’s VAS scores were considerably higher than those of the proximal and mid groups (e.g., 8th hour: 40.69 ± 10.50 vs. 26.94 ± 8.72 and 23.47 ± 7.64, *p* < 0.001), whereas there was no difference between the proximal and mid groups (*p* > 0.05). At 24 h, the distal group had significantly higher functional pain scores on all dynamic and static measures (*p* < 0.001). The proximal and mid groups had longer sensory block durations (5.38 ± 1.33 and 5.03 ± 1.29 h) than the distal group (2.95 ± 0.95 h, *p* < 0.001). While mid-level ACB maintained motor function, 13.9% of patients receiving proximal ACB experienced transient motor block. **Conclusions:** After TKA, injection level has a major impact on postoperative analgesia. Mid-level ACB supports the employment of multimodal analgesia techniques by offering the best possible compromise between efficient pain management and motor function maintenance.

## 1. Introduction

### Background/Rationale

One of the most commonly done orthopedic operations in worldwide practice, total knee arthroplasty (TKA), is frequently accompanied by significant postoperative pain, which poses a significant obstacle to early mobilization, functional recovery, and improved recovery pathways. Prolonged hospital stays, higher opioid use, delayed rehabilitation, and the possibility of developing chronic postoperative pain have all been associated with inadequate pain management. Therefore, a crucial part of perioperative care in TKA is maximizing postoperative pain control [1,2].

It has been demonstrated that postoperative pain intensity varies significantly, even when the degree of tissue damage appears to be similar across patients. Most patients have been shown to have moderate-to-severe pain, especially in the first 48 h after surgery. It has been demonstrated that pain-related discomfort and distress negatively impact the entire rehabilitation process and are linked to a delayed start of rehabilitation [3].

A variety of regional analgesic methods, such as intra-articular local anesthetic injections and peripheral nerve blocks like femoral, obturator, sciatic, and adductor canal blocks, are frequently used for postoperative pain management after total knee replacement. Although the femoral nerve block has long been considered the “gold standard,” early mobilization may be hampered by its correlation with quadriceps decreased muscle strength. Alternative regional procedures have been actively investigated because of these restrictions as well as the difficulties experienced during patient mobilization and rehabilitation. Among these, the adductor canal block has shown promise as a postoperative analgesic with low degradation of quadriceps muscular strength, and it has been the subject of more research in recent years. Due to its primarily sensory blocking and no effect on quadriceps function, adductor canal block (ACB) has become widely accepted as a component of multimodal analgesia treatments. The rising use of ACB in enhanced recovery after surgery (ERAS) protocols is supported by this motor-sparing feature that sets it apart from femoral nerve block [4].

The adductor canal is not a uniform anatomical structure but rather a dynamic anatomical corridor with considerable neural heterogeneity. The anatomical complexity of the adductor canal provides the primary rationale for investigating different injection levels. The canal extends from the apex of the femoral triangle to the adductor hiatus and contains several neural structures contributing to knee joint innervation. Although the saphenous nerve is the principal sensory nerve within the canal, the nerve to vastus medialis and articular branches originating from the obturator nerve demonstrate variable courses along the proximal-to-distal extent of the canal. Cadaveric and imaging studies have shown that local anesthetic spread may differ substantially depending on the injection location, potentially influencing the extent of articular branch blockade and postoperative analgesic efficacy [5,6].

Anatomical investigations have further demonstrated that proximal injections may facilitate spread toward the distal femoral triangle and branches supplying the anteromedial knee capsule, whereas distal injections may predominantly affect the saphenous nerve distribution. Consequently, injection level may alter not only the quality and duration of sensory blockade but also the likelihood of unintended motor involvement through spread to branches associated with the vastus medialis muscle. These anatomical considerations suggest that the adductor canal should not be regarded as a uniform target and support the need for comparative clinical evaluation of different injection levels [7,8].

In terms of postoperative pain levels, analgesic efficacy, and the degree of motor blockade, prior research has thoroughly compared femoral nerve block and adductor canal block, both used for postoperative analgesia after knee surgery. In order to maximize postoperative pain management, studies assessing continuous catheter implantation into the adductor canal have also been published. The ideal injection level within the adductor canal is still unclear despite ACB’s extensive clinical use. While direct comparisons between various injection levels within the adductor canal under standardized conditions are still rare, the majority of current research has concentrated on comparisons between ACB and femoral nerve block or on continuous catheter approaches.

In order to compare the effects of proximal, mid, and distal ACB on postoperative analgesic efficacy, pain levels under both static and dynamic conditions, motor and sensory block characteristics, and opioid consumption in patients undergoing TKA, the current study was designed.

This study’s main goal was to ascertain whether the adductor canal block injection level affects postoperative analgesic effectiveness and pain experienced during functional activities after total knee replacement. Assessing the impact of injection level on motor function preservation, sensory block length, and opioid use were secondary goals.

We hypothesized that different adductor canal injection levels would result in clinically meaningful differences in postoperative analgesia, sensory block characteristics, opioid consumption, and motor function preservation following total knee arthroplasty.

## 2. Materials and Methods

### 2.1. Study Design

This study was planned as a prospective, observational study comparing three predefined intervention groups with approval from the Erzincan Binali Yildirim University Clinical Research Ethics Committee (Approval date: 16 November 2023; Approval number: 2023-20/05). The study was conducted in accordance with the principles of the Declaration of Helsinki. This manuscript was prepared and reported in accordance with the Strengthening the Reporting of Observational Studies in Epidemiology (STROBE) guidelines recommended by the EQUATOR Network.

### 2.2. Setting

The study was conducted at Erzincan Binali Yildirim University Mengucek Gazi Training and Research Hospital between November 2023 and November 2024. All procedures were performed in a tertiary care academic hospital setting.

### 2.3. Participants

A total of 108 patients scheduled for unilateral primary total knee arthroplasty under spinal anesthesia were included.

### 2.4. Criteria for Inclusion

- Age 18 to 70;
- Physical status II–III of the American Society of Anesthesiologists (ASA);
- Receiving spinal anesthesia during primary unilateral TKA;

### 2.5. Exclusion Criteria

- ASA I or ≥IV;
- Revision or bilateral TKA;
- General anesthesia;
- Neuromuscular disorders;
- History of local anesthetic toxicity;
- Coagulopathy or bleeding disorders;
- Refusal to participate.

One day before surgery, all patients who were planned to take part in the trial received comprehensive information about the study protocol. Written informed permission was obtained after the procedures were explained verbally and in writing.

Patients were moved to the operating room on the morning of the procedure, when routine American Society of Anesthesiologists (ASA) monitoring was used. This included body temperature monitoring, electrocardiography, noninvasive blood pressure measurement, and oxygen saturation (SpO_2_). All patients received 15–16 mg of 0.5% hyperbaric bupivacaine via the L3–L4 or L4–L5 intervertebral region during spinal anesthesia. Sensory block level was tested using the pin-prick test, and patients displaying bilateral sensory loss at the T10 dermatome level along with a Bromage score of ≥3 were considered ready for operation. Blood pressure, heart rate, and SpO_2_ were all constantly monitored during surgery. Following surgery, the pin-prick test was used to reevaluate the degree of sensory block, and the Bromage scale was used to measure motor block.

Patients were placed supine with their hips externally rotated at the end of the procedure while they were still on the operating table. Povidone-iodine solution was used to disinfect the block location. An Esaote MyLabTM Six ultrasound (Esaote S.p.A., Genoa, Italy) system with an SL1543 3–13 MHz linear probe was used to execute ultrasound-guided blocks. A 22-gauge, 80-mm peripheral nerve block needle (Stimuplex^®^ Ultra 360^®^, 22 G × 80 mm, 30° beveled, and echogenic insulated needle with extension set, (B. Braun Melsungen AG, Melsungen, Germany) was used for all blocks using the in-plane technique. Initially, the linear ultrasound probe was positioned at the inguinal crease. Following the Sartorius muscle from the distal femoral triangle’s apex, the probe was moved distally when the femoral artery was located.

### 2.6. Group Allocation

Patients were included into three groups based on the level of adductor canal block performed: proximal (Group I), mid (Group II), and distal (Group III). Inclusion was performed prior to block administration according to a predefined and standardized institutional protocol. The protocol determined the block level before postoperative outcome assessment and was not based on patient preference, postoperative pain status, or intraoperative outcomes. No randomization procedure was performed. All blocks were performed by anesthesiologists with at least five years of experience in peripheral nerve block techniques who were not involved in postoperative follow-up assessments.

No randomization was performed, and group allocation was based on a predefined protocol, which may introduce potential selection bias. However, baseline demographic and clinical characteristics were comparable between groups.

### 2.7. Variables

#### 2.7.1. Primary Outcome

- Postoperative pain intensity (VAS scores at 1, 4, 8, 12, and 24 h).

#### 2.7.2. Secondary Outcomes

- Motor block presence and duration;
- Sensory block duration;
- Opioid consumption (tramadol, mg/24 h);
- Pain during standardized static and dynamic functional tests at 24 h.

All blocks were performed using an ultrasound-guided in-plane approach. The needle tip was positioned beneath the subsartorial fascia and lateral to the saphenous nerve adjacent to the femoral artery. Needle repositioning was performed when necessary to ensure adequate spread of local anesthetic around the saphenous nerve and throughout the subsartorial compartment.

**Figure 1:**
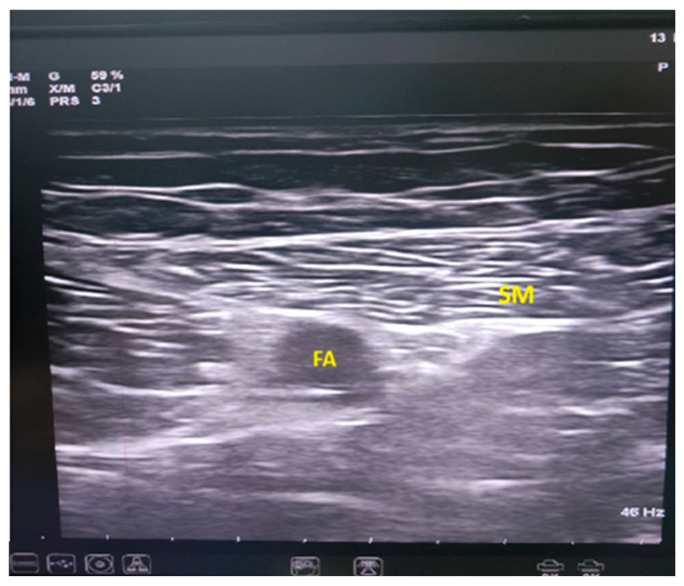
Ultrasound Image of the Proximal Adductor Canal Block (FA: Femoral artery, SM: Sartorius muscle).

The proximal adductor canal level (Figure 1) is the intersection of the medial borders of the adductor longus and Sartorius muscles. Following a negative aspiration, 30 milliliters (mL) of 0.25% bupivacaine was injected around the saphenous nerve, spreading under ultrasonography to the subsartorial area. To reduce the possibility of intravascular administration, negative aspiration was repeated every 5 mL of injection.

**Figure 2:**
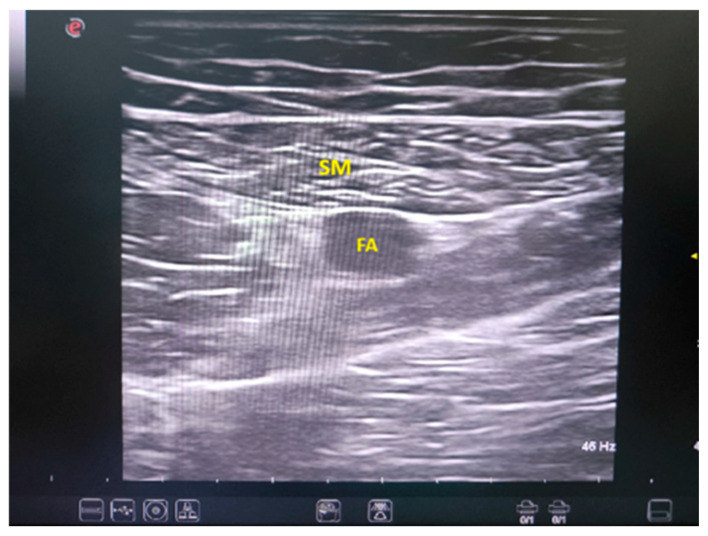
Ultrasound Image of the Mid Adductor Canal Block (FA: Femoral artery, SM: Sartorius muscle).

The area where the femoral artery and the Sartorius muscle run parallel to one another is referred to as the mid-adductor canal block level (Figure 2). After a negative aspiration, the saphenous nerve was surrounded by an injection of 30 mL of 0.25% bupivacaine, which was confirmed by ultrasound to have spread into the subsartorial space. To reduce the possibility of intravascular administration, negative aspiration was repeated every 5 mL of injection.

**Figure 3:**
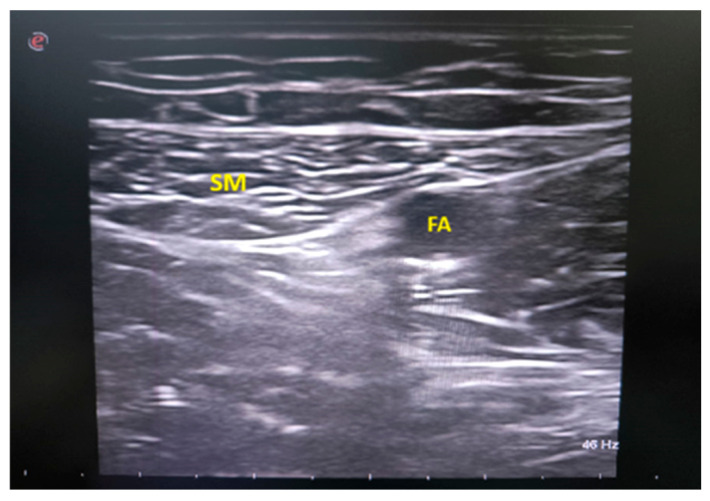
Ultrasound Image of the Distal Adductor Canal Block (FA: Femoral artery, SM: Sartorius muscle).

The Sartorius muscle was traced distally while the ultrasonic probe was moved in the direction of the patella’s superior pole to determine the distal adductor canal level (Figure 3). The femoral artery’s deeper path and proximity to the adductor hiatus were identified as this level. After a negative aspiration, the saphenous nerve was surrounded by an injection of 30 mL of 0.25% bupivacaine, which was confirmed by ultrasound to have spread into the subsartorial space. To reduce the possibility of intravascular administration, negative aspiration was repeated every 5 mL of injection.

### 2.8. Data Collection and Measurements

Standardized patient follow-up forms were used to systematically capture preoperative anesthetic evaluation forms, intraoperative recorded data, and all parameters evaluated during postoperative follow-up visits during the first 24 h. A 100-mm Visual Analog Scale (VAS), with 0 representing no pain and 100 representing the highest possible painful experience, was used to measure postoperative pain severity at 1, 4, 8, 12, and 24 h.

Postoperative analgesia was standardized across all groups. All patients received a uniform multimodal analgesic regimen consisting of intravenous nonsteroidal anti-inflammatory drugs (NSAIDs) administered at regular intervals. Rescue analgesia was provided with intravenous tramadol upon patient request or when the visual analog scale score exceeded 40 mm. The type, dose, and timing of all administered analgesics were recorded.

Following the adductor canal block, the presence or absence of motor and sensory block was assessed, and if present, the time to block resolution was recorded. Motor block was evaluated using the patient’s ability to perform an active straight leg raise against gravity. Although no formal motor scoring system was applied, this assessment reflects a clinically relevant measure of quadriceps function commonly used in postoperative practice. Sensory block was assessed using pin-prick testing along the saphenous nerve distribution, extending from the medial aspect of the knee to the mid-medial calf. At the time of sensory assessment, it was confirmed that the corresponding VAS score was <45 mm.

Additionally, a total of seven standardized tests—three dynamic and four static functional tests—were used to gauge the patients’ mobilization capacity 24 h after surgery. The appropriate VAS score was noted in the patient follow-up forms following each test. The same doctor, who was blind to the type of nerve block used, did postoperative evaluations.

### 2.9. Tests Assessed During Active (Dynamic) Movements

(A1) While in the supine position, the patient was instructed to slowly flex the knee as much as possible without lifting the heel from the bed and then return to the initial position. Pain intensity during this movement was assessed and scored using the Visual Analog Scale (VAS).

(A2) The Timed Up and Go (TUG) test measures the time required for the patient to stand up from a chair, walk 3 m, and return to the chair. Pain intensity experienced during this task was evaluated and recorded using the VAS.

(A3) The Five Times Sit-to-Stand Test (5 × SST) measures the time required for the patient to transition from a full sitting position to a full standing position five consecutive times. Pain intensity during this activity was assessed using the VAS.

### 2.10. Tests Assessed During Passive (Static) Movements

(P1) Straight Leg Raise (SLR) Test: The patient was positioned supine without a pillow under the head. The hip was placed in medial rotation and adduction, with the knee in full extension. While maintaining the knee in extension, the examiner lifted the leg by holding the posterior aspect of the ankle. Pain intensity during this maneuver was assessed using the VAS.

(P2) While in the supine position, a pillow was placed under the ankle, and the patient was instructed to maintain the knee in full extension for 2 min. Pain intensity during this period was evaluated using the VAS.

(P3) A towel roll was placed between the patient’s legs, and the patient was instructed to compress the towel using both legs and maintain this position for 15 s. Pain intensity during this maneuver was assessed using the VAS.

(P4) The patient was positioned in an upright sitting posture with the legs dangling. After maintaining this position for 15 s, pain intensity during this period was assessed using the VAS.

To ensure standardization and reproducibility, all functional assessments were performed according to a predefined testing sequence in all patients. Static tests were performed first (P1–P4), followed by dynamic tests (A1–A3). A resting interval of 2 min was allowed between consecutive tests to minimize the influence of fatigue and carry-over pain effects.

### 2.11. Bias

To minimize bias, the anesthesiologist performing the block was not involved in postoperative assessments. Outcome assessors, surgeons, nursing staff, and patients were blinded to group allocation.

Blinding was ensured by performing the adductor canal block after completion of surgery. Surgeons and nursing staff were not present during block administration, thereby preventing awareness of group allocation. The anesthesiologist responsible for block application was not involved in postoperative outcome evaluation.

Due to the nature of the intervention, complete double blinding was not feasible. Therefore, the study was conducted in an assessor-blinded design.

Selection bias could not be completely excluded due to the non-randomized design; however, baseline characteristics were comparable between groups. Performance bias was minimized by standardizing perioperative management, and detection bias was reduced through blinded outcome assessment.

### 2.12. Statistical Analysis

Statistical analyses were performed using IBM SPSS Statistics for Windows, Version 25.0 (IBM Corp., Armonk, NY, USA).

Sample size estimation was performed based on a two-way mixed-design ANOVA. Assuming a type I error rate (α) of 0.05 and a statistical power (1 − β) of 0.80, with three study groups and five repeated measurement time points, and considering a medium effect size (Cohen’s f = 0.25) and a correlation coefficient of 0.50 among repeated measures, the minimum required sample size was calculated as 32 participants per group, corresponding to a total of 96 patients. Considering potential data loss, the total sample size was increased to 108 patients.

Sample size estimation was based on a medium effect size (Cohen’s f = 0.25), consistent with Cohen’s conventional classification for ANOVA models, in the absence of sufficiently robust prior data regarding differences among three adductor canal injection levels.

Descriptive statistics were presented as mean ± standard deviation, median (minimum–maximum), and categorical variables as frequencies and percentages. Normality of continuous variables was assessed using the Shapiro–Wilk test and visual inspection of histograms. Homogeneity of variances was assessed using Levene’s test.

The significance of differences in mean values between groups was evaluated using one-way analysis of variance (ANOVA). Pairwise comparisons were performed using Tukey’s post hoc test. Categorical variables were analyzed using Pearson’s chi-square test.

Changes over time within and between groups were assessed using a two-way mixed-design ANOVA, with group as the between-subject factor and time as the within-subject factor.

A two-sided *p*-value < 0.05 was considered statistically significant. For continuous outcomes, pairwise comparisons are reported as mean differences (MDs) with corresponding 95% confidence intervals (CIs).

## 3. Results

### 3.1. Participant Flow

**Figure 4:**
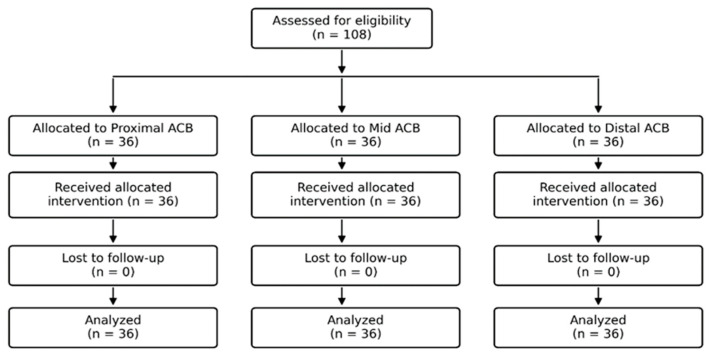
Flow diagram illustrating the group allocation, follow-up, and analysis of the study population.

A total of 108 patients were assessed for eligibility and all were included in the study. No patients were excluded after allocation, and no losses to follow-up or protocol deviations occurred during the study period. All enrolled patients completed the 24-h follow-up and were included in the final analysis (Figure 4).

### 3.2. Baseline Characteristics

The study population consisted of 108 patients, of whom 91 (84.3%) were female and 17 (15.7%) were male. The distribution of patients across the three groups was equal (*n* = 36 per group).

There were no statistically significant differences among the groups in terms of age, sex distribution, body mass index (BMI), ASA physical status, or duration of surgery (*p* > 0.05 for all comparisons). These findings indicate that the groups were comparable at baseline (Table 1).

### 3.3. Postoperative Pain Scores

**Figure 5:**
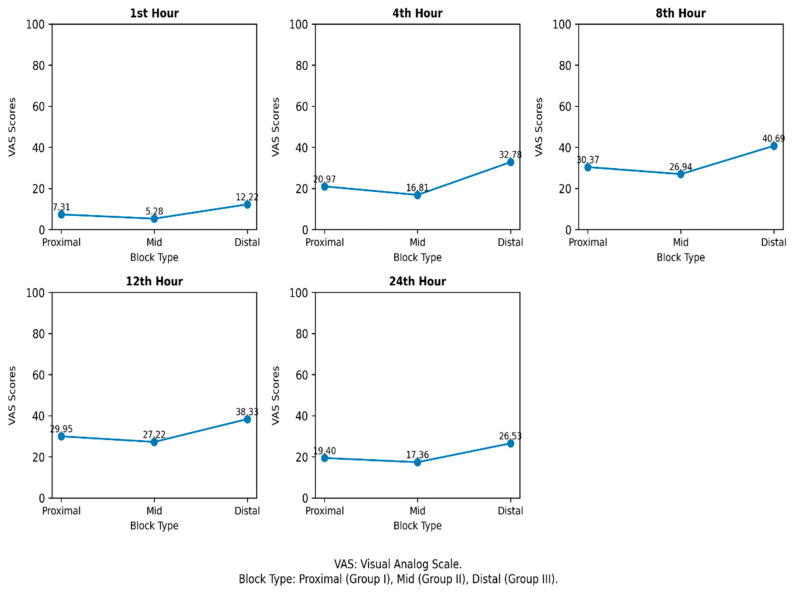
VAS scores of the groups at the 1st, 4th, 8th, 12th, and 24th postoperative hours.

Postoperative VAS scores at 1, 4, 8, 12, and 24 h are summarized in Table 2 and illustrated in Figure 5.

At all postoperative time points, the distal ACB group (Group III) had significantly higher VAS scores than the proximal (Group I) and mid (Group II) groups (*p* < 0.001 for all comparisons), with peak values noted at 8 and 12 h postoperatively.

At any times point, Group I and Group II’s VAS scores did not differ statistically significantly (all *p* > 0.05).

These differences showed both statistical significance and clinical relevance because they were more than the minimum clinically important difference (MCID) for VAS pain scores. On the VAS, which is widely recognized as the cutoff point for clinical significance, the observed differences between groups were more than 10 mm.

**Figure 6:**
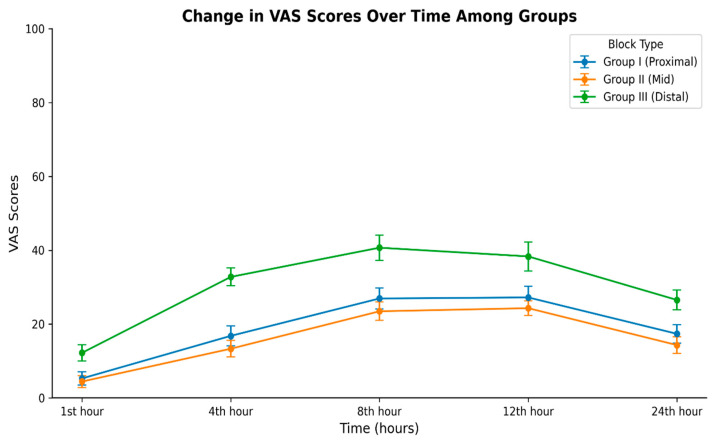
Change in VAS scores over the time among groups following total knee arthroplasty. VAS scores at the 1st, 4th, 8th, 12th, and 24th postoperative hours are shown. Error bars represent 95% confidence intervals.

A two-way mixed ANOVA demonstrated significant main effects of time (*p* < 0.001) and group (*p* < 0.001), as well as a significant time × group interaction (*p* < 0.001), indicating that the trajectory of VAS scores over time differed significantly among the groups, with Group III demonstrating persistently higher pain scores and a more pronounced peak at 8–12 h postoperatively (Figure 6).

### 3.4. Functional Pain Assessment at 24 h

**Figure 7:**
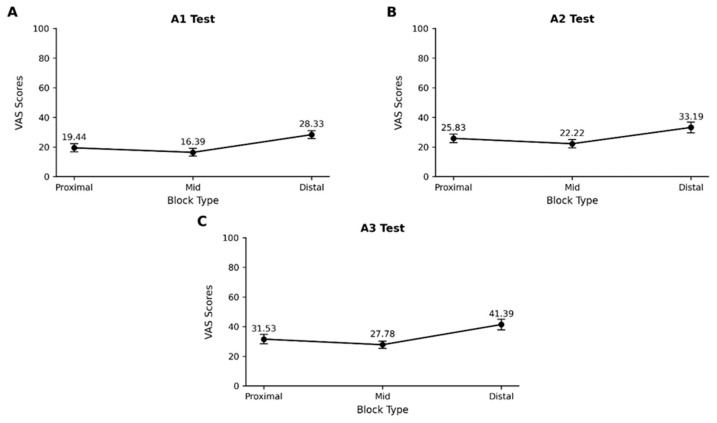
Distribution of VAS scores across groups during dynamic movement tests at the 24th postoperative hour. (**A**) A1 Test, (**B**) A2 Test, and (**C**) A3 Test. VAS scores are presented for proximal, mid, and distal adductor canal block groups. Error bars present 95% confidence intervals.

**Figure 8:**
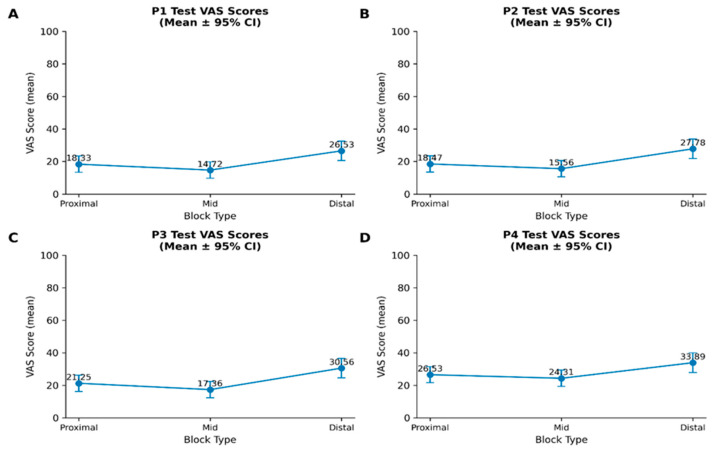
Distribution of VAS Scores across Groups during Static Movement Tests at the 24th Postoperative Hour. (**A**) P1 Test, (**B**) P2 Test, (**C**) P3 Test, and (**D**) P4 Test. VAS scores are presented as mean ± 95% confidence interval (CI) for the proximal, mid, and distal adductor canal block groups.

Pain scores during dynamic and static functional tests performed at postoperative 24 h are presented in Table 3 and Table 4 and Figure 7 and Figure 8.

Across all dynamic (A1–A3) and static (P1–P4) tests, VAS scores were significantly higher in Group III compared to Groups I and II (*p* < 0.001 for all comparisons).

In overall functional pain assessment, there were no statistically significant differences between Group I and Group II (*p* > 0.05).

These findings demonstrate a clinically significant increase in pain during functional activities in the distal ACB group compared to Groups I and II, particularly during dynamic activities.

### 3.5. Motor Block

Transient motor block was observed in 5 patients (13.9%) in Group I, with a duration of 5–6 h; no motor block was detected in Groups II and III, supporting the motor-sparing profile of mid and distal ACB.

### 3.6. Sensory Block Duration

**Figure 9:**
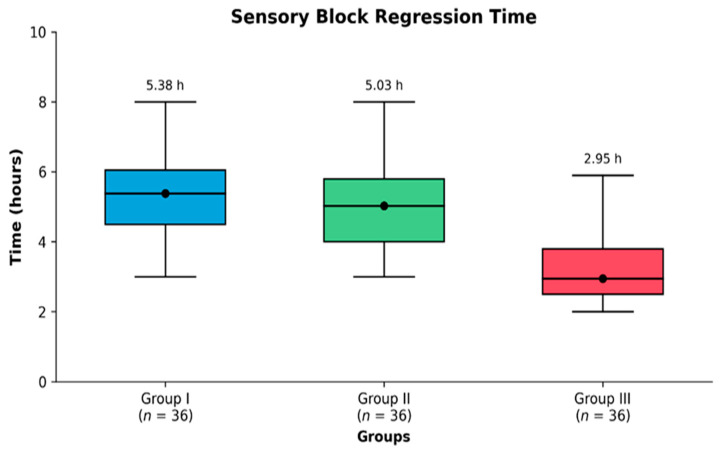
Distribution of Sensory Block Regression Time among Groups. Boxplots indicate median (horizontal line), interquartile range (box), and minimum-maximum values (whiskers). The black dot represents the mean value. Group III (distal adductor canal block) demonstrated a significantly shorter sensory block regression time compared with Group I and Group II (*p* < 0.001).

Sensory block duration differed significantly among the groups (Table 5 and Figure 9).

Groups I and II had considerably longer sensory block durations than Group III (*p* < 0.001 for both comparisons).

No statistically significant difference was observed between Group I and Group II (*p* = 0.442).

### 3.7. Opioid Consumption

Total tramadol consumption over the first 24 h was significantly higher in Group III compared to Groups I and II (*p* < 0.001), with mean values of 80.56 ± 49.68 mg in Group I, 75.00 ± 56.70 mg in Group II, and 141.67 ± 45.51 mg in Group III.

No statistically significant difference was observed between Group I and Group II (*p* = 0.888) (Table 6). Consistent with the observed pain scores, tramadol consumption was numerically lower in Group II compared to Group I.

**Figure 10:**
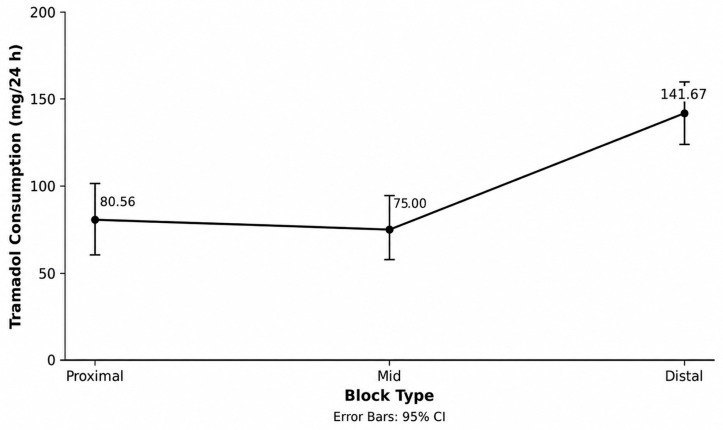
Tramadol consumption across groups within the first 24 h following total knee arthroplasty. Values are presented as mean ± 95% confidence interval (CI). The distal adductor canal block group demonstrated significantly higher opioid consumption compared with the proximal and mid groups (*p* < 0.001).

Figure 10, Table 6 and Table 7 provide an overview of the tramadol consumption during the 24 h following surgery.

Additional NSAID requirements were also evaluated during the first 24 postoperative hours. NSAID use was observed in 10 patients (27.8%) in Group I, 14 patients (38.9%) in Group II, and 13 patients (36.1%) in Group III. Mean NSAID consumption was 13.6 ± 24.9 mg, 17.2 ± 26.0 mg, and 25.0 ± 36.8 mg in Groups I, II, and III, respectively, with no statistically significant difference among the groups (*p* = 0.509).

**Figure 11:**
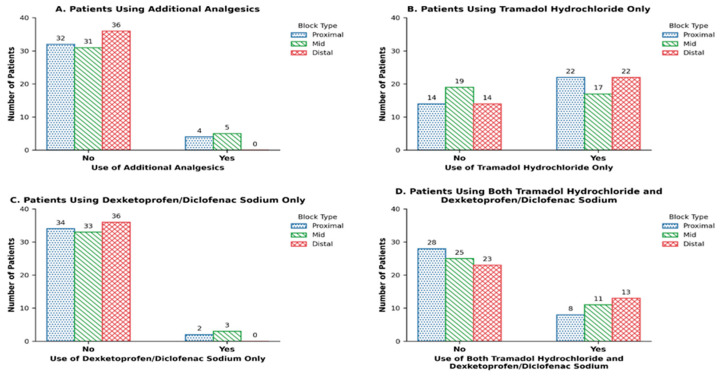
Comparison of rescue analgesic requirements among proximal, mid, and distal adductor canal block groups during the first 24 postoperative hours.

Additionally, the requirement for supplemental NSAID analgesia was greater in Group III compared to Groups I and II. The number of patients requiring additional analgesics and the corresponding consumption differences are summarized in Figure 11.

### 3.8. Complications

No block-related complications were observed. Specifically, there were no cases of local anesthetic systemic toxicity (LAST), vascular injury, hematoma, inadvertent intravascular injection, infection, or allergic reactions.

### 3.9. Summary of Main Findings

This prospective study shows that postoperative analgesic effects after total knee arthroplasty are highly influenced by the amount of injection within the adductor canal. Distal ACB was consistently associated with inferior analgesic efficacy, as evidenced by higher VAS scores across all time points, increased opioid requirements, and a shorter duration of sensory blockade.

In contrast, proximal and mid-level ACB provided comparable pain control; however, transient motor block occurred exclusively in the proximal group, indicating a potential trade-off between analgesic spread and motor involvement.

Importantly, mid-level ACB demonstrated analgesic outcomes comparable to proximal ACB while avoiding the transient motor block observed in the proximal group. These findings suggest that mid-level injection may provide a favorable balance between analgesic efficacy and motor preservation. However, given the observational design of the present study, randomized controlled trials are required to confirm these findings and determine the optimal injection level for adductor canal block following TKA.

## 4. Discussion

### 4.1. Key Findings

In this prospective observational study, we evaluated the effects of proximal, mid, and distal adductor canal block (ACB) injection levels on postoperative analgesia, motor and sensory block features, and opioid consumption following total knee arthroplasty (TKA).

The main conclusions can be summed up as follows. At all-time points, including functional evaluations, distal ACB was linked to noticeably greater pain levels, suggesting lower analgesic efficacy. On the other hand, compared to distal ACB, proximal and mid ACB offered similar analgesic effects while requiring fewer opioids. Only the proximal group experienced motor block, while mid-level ACB maintained effective analgesia while preserving motor function. The distal group likewise experienced a shorter sensory block duration.

Overall, these results suggest that an important factor influencing both analgesic efficacy and motor preservation is the injection level within the adductor canal. Clinically speaking, mid-level ACB seems to offer the best balance between maintaining quadriceps function and effectively controlling pain, which supports its usage in improved recovery procedures.

### 4.2. Comparison with Existing Literature

Compared to femoral nerve block, prior research has shown that adductor canal block offers efficient postoperative analgesia while maintaining quadriceps strength. The ideal injection level inside the adductor canal, however, is yet unknown. Our results add to the existing literature by demonstrating that injection level is associated with postoperative analgesic outcomes and pain experienced during functional activities following total knee arthroplasty.

Distal injections may cause insufficient dissemination to articular branches, resulting in inferior analgesia, in accordance with anatomical studies that demonstrate varied distribution of sensory and motor nerve branches along the adductor canal. On the other hand, branches that contribute to quadriceps motor function may have more proximal spread, which could account for the temporary motor block seen in the proximal group. By limiting unwanted motor engagement and offering adequate sensory coverage, the mid-level injection seems to strike a balance between these two extremes.

### 4.3. Analgesic Efficacy

Severe early postoperative pain following total knee arthroplasty (TKA) has a direct impact on patient satisfaction, rehabilitation, and mobilization. A significant percentage of patients have moderate-to-severe pain, and poor management has been connected to higher opioid consumption, delayed mobilization, and the emergence of chronic pain [9]. Peripheral nerve blocks are consequently a crucial part of multimodal analgesia, which is the standard of therapy [10]. Although it can effectively relieve pain while maintaining quadriceps strength, adductor canal block (ACB) has become widely accepted among these, especially in Enhanced Recovery after Surgery (ERAS) protocols [1,2].

Higher pain scores following distal ACB suggest inadequate anteromedial knee innervation coverage. This probably represents the adductor canal’s segmental anatomical structure. Knee innervation is influenced by the saphenous nerve, nerve to vastus medialis, and obturator nerve branches, which diverge along the proximal-to-distal route [6]. Our results are consistent with those of Burckett-St. Laurant et al., who showed that proximal injections offer wider analgesic coverage by affecting not only the saphenous nerve but also the nerve to the vastus medialis and obturator branches [7].

Distal injections probably impact a more constrained sensory domain, but proximal injections might span a larger brain distribution. Inadequate blocking could result from inadequate coverage of pertinent branches. As a result, there is less pain management, which raises the need for opioids.

These results are corroborated by imaging and cadaveric research. According to Runge et al., proximal ACB improves blockage of the anteromedial knee capsule by facilitating dissemination to genicular nerve branches [5]. Similarly, Johnston et al. and Tran et al. reported greater longitudinal spread of local anesthetic following proximal injections [6,8].

Studies evaluating single-shot ACB have produced heterogeneous findings. Romano et al. reported superior analgesic performance with proximal ACB compared with distal application [11], whereas a meta-analysis by Lukai Zhang et al. found no significant difference between proximal and distal approaches [12]. In contrast, Qiangqiang Li et al. reported superior early postoperative analgesia with proximal injections [13].

Continuous catheter studies should be interpreted separately because local anesthetic spread may be influenced by prolonged infusion. Yuda Fei et al. compared proximal and mid-level continuous ACB following TKA and found no difference in NRS scores [14]. However, unlike the present study, their investigation did not include functional pain assessments such as the Timed Up and Go (TUG) test or Five Times Sit-to-Stand Test, which may provide a more comprehensive evaluation of postoperative recovery. Likewise, Mariano et al. demonstrated a modest advantage of proximal ACB without increased motor blockade in patients receiving continuous catheter techniques [15]. Therefore, direct comparison between continuous catheter studies and the present single-shot ACB study should be interpreted with caution.

Methodological variability is probably the cause of these differences. Block performance is significantly influenced not just by injection dose but also by local anesthetic amount, concentration, and technique (single-shot vs. continuous catheter). Higher volumes and continuous methods could improve spread and conceal level-dependent variations.

The single-injection technique may have reduced these confounding effects in the current investigation, enabling more distinct level distinction. It’s likely that the comparatively high volume (30 mL of 0.25% bupivacaine) encouraged cranio-caudal spread, especially at the mid-level, leading to more uniform covering. Additionally, ACB seems to act like a fascial plane block, where spread to nearby neuronal regions is facilitated by adequate volume. This finding lends more credence to the idea that volume-dependent hydrodissection and injection level both influence cranio-caudal distribution.

Both proximal and mid-levels appear to have sufficient sensory coverage because there is no difference in pain scores between them. Nonetheless, the midgroup’s reduced opioid consumption is clinically significant. This finding suggests a more selective neural blockade. Anatomically, the saphenous nerve, the nerve to the vastus medialis, and the obturator branches all contribute to the mid-adductor canal. This area might serve as a “convergence zone,” maximizing analgesic distribution while reducing motor involvement.

### 4.4. Functional Pain and Rehabilitation-Relevant Outcomes

#### Pain During Static and Dynamic Movement

Both resting and movement-related pain were thoroughly assessed in this study. Optimal control of functional pain was demonstrated by proximal and mid-level ACB, which produced considerably lower pain scores than distal ACB across several static and dynamic assessments at 24 h. This observation has clinical significance since movement-related pain has a direct impact on rehabilitation [16].

Shah et al. examined the effects of femoral nerve block and adductor canal block on ambulation, pain ratings, and opioid consumption after total knee arthroplasty. Timed Up and Go (TUG), 10-m walk, 30-s chair stand test, time to active straight leg lift, and ambulation distance were among the ambulation-related outcomes that were significantly improved in the ACB group (*p* < 0.001). Compared to femoral nerve block, ACB did not offer better pain relief, despite improving ambulation and facilitating an earlier functional recovery [17].

Chaiperm et al. reported no differences in pain during knee extension and flexion between groups [18]. In contrast, Greenky et al. found greater active flexion at 24 h in the proximal ACB group, although this difference was not sustained at 6 weeks [19].

The majority of earlier research has mostly examined pain at rest, with little attention paid to pain associated with movement. The current study offers a more thorough examination of clinically important pain outcomes by combining both static and dynamic evaluations.

### 4.5. Motor Function Preservation

Preservation of quadriceps function is essential for early mobilization and fall prevention. Transient motor block occurred exclusively in the proximal ACB group, with an incidence of 13.9%, consistent with the 10–15% range reported by Jaeger et al. [20]. Similarly, Abdallah et al. reported comparable reductions in quadriceps strength across ACB groups (16.7%, 13.4%, and 15.3%, respectively) [21].

In our study, motor block resolved by the 5th postoperative hour in two patients and by the 6th hour in the remaining three. Adıyeke et al. reported a longer duration of motor block in patients receiving ACB compared to those without ACB (367.3 vs. 227.5 min), consistent with our findings [22].

ACB primarily targets the anteromedial sensory innervation of the knee by blocking the saphenous nerve and obturator branches medial to the femoral artery. As motor fibers are largely spared, quadriceps strength is generally preserved, facilitating early mobilization [10,21]. However, the influence of injection level on the balance between sensory and motor blockade remains incompletely understood [23].

Our findings suggest that proximal injections may carry a higher risk of spread to motor branches, likely due to their proximity to the femoral triangle. On the other hand, a more selective sensory blockage at these levels is supported by the lack of motor block in the mid and distal groups. This distinction is clinically important in settings where early mobilization and fall prevention are priorities, suggesting that mid-level ACB may offer a meaningful advantage in terms of motor-sparing analgesia.

In addition, the study was powered primarily for repeated-measures pain outcomes and may have been underpowered to detect differences in relatively infrequent secondary outcomes, such as transient motor block.

### 4.6. Sensory Block Duration

Adıyeke et al. reported a longer sensory block duration in patients receiving ACB compared to those without ACB (337 ± 159 vs. 206 ± 107 min) [22]. In our study, sensory block duration was 480 min in both the proximal and mid groups and 360 min in the distal group.

More efficient local anesthetic diffusion at these levels is suggested by the extended duration seen in the proximal and mid groups. This could be explained by improved cranio-caudal distribution in the adductor canal, especially when injection volumes are higher.

Previous studies indicate that volumes of 20–30 mL facilitate extensive spread and improve block performance [17,24]. Consistent with this, Tran et al. demonstrated that proximal injections can reach a broader neural network in cadaveric models [6].

The use of a standardized volume across all groups in our study enabled a more objective comparison of injection level, minimizing volume-related confounding effects.

### 4.7. Opioid Consumption

Opioid consumption is a clinically important outcome. In our study, 24-h tramadol use was 80.56 ± 49.68 mg in Group I, 75.00 ± 56.70 mg in Group II, and 141.67 ± 45.51 mg in Group III. The distal group required significantly more tramadol, indicating inadequate pain control and a higher risk of opioid-related adverse effects [1].

Previous studies have demonstrated that ACB has an opioid-sparing impact, especially when it comes to early mobilization and patient satisfaction. In a randomized study of 108 patients undergoing anterior cruciate ligament reconstruction, Abdallah et al. reported 24-h morphine consumption of 34.3 ± 19.1 mg, 64.0 ± 33.6 mg, and 65.7 ± 22.9 mg in the proximal, mid, and distal groups, respectively [21]. Similarly, Schnabel et al. demonstrated in a meta-analysis that ACB reduces opioid consumption by approximately 35% while providing analgesia comparable to femoral nerve block [25]. Notably, this opioid-sparing effect appears to diminish with distal applications [26].

Taken together, proximal and particularly mid-level ACB provide effective pain control while reducing opioid requirements. This balance represents a clinically advantageous strategy that may be readily translated into practice.

### 4.8. Clinical Implications

Although broadly consistent with the existing literature, the limited number of studies directly comparing injection levels within the adductor canal underscores the originality of our findings. A comparatively unexplored area of study is the comparison of three different levels utilizing a single-injection approach. Additionally, this study’s clinical significance is improved by the evaluation of both functional and resting pain.

The amount of injection in the adductor canal affects motor performance, opioid use, and pain management. Mid-level ACB facilitates early mobilization and supports adherence to ERAS protocols by offering sufficient postoperative analgesia while reducing the risk of motor blockage. For this reason, it might be seen as a better standard technique in clinical practice.

These results directly affect ERAS pathways, where early ambulation, fewer postoperative complications, and quicker functional recovery depend on striking the greatest balance between analgesia and quadriceps function preservation.

### 4.9. Limitations

This study has several limitations that should be acknowledged. First, the non-randomized design may introduce selection bias and residual confounding, despite comparable baseline characteristics between groups. Second, this was a single-center study, which may limit the generalizability of the findings. Third, the follow-up period was limited to 24 h, precluding assessment of long-term pain outcomes and functional recovery. Finally, motor function was evaluated using clinical assessment rather than objective quantitative methods such as dynamometry. Future multicenter studies with randomized designs, longer follow-up, and objective assessment tools are needed to confirm these findings.

### 4.10. Generalisability (External Validity)

Despite these limitations, this study has several notable strengths. It employed a prospective design with a homogeneous patient population managed by a single surgical team, alongside standardized anesthesia and analgesia protocols and an adequate sample size.

Both resting and movement-related pain were comprehensively assessed, providing a multidimensional evaluation of clinically relevant outcomes. In addition, clear anatomical standardization of proximal, mid, and distal adductor canal levels using predefined landmarks ensured consistency in block application.

The simultaneous evaluation of motor and sensory block characteristics further strengthens the clinical relevance of the findings. Moreover, the direct comparison of three different ACB levels—an area addressed in only a limited number of studies—represents a novel contribution to the literature.

## 5. Conclusions

In conclusion, postoperative analgesic outcomes and pain during functional activities following total knee arthroplasty were associated with the injection level used for adductor canal block. Distal injections were associated with higher pain scores and greater opioid requirements, whereas proximal injections were associated with a small incidence of transient motor block. Mid-level adductor canal block demonstrated analgesic outcomes comparable to proximal injection while avoiding the motor block observed in the proximal group. These findings suggest that mid-level ACB may represent a favorable balance between analgesic efficacy and motor preservation; however, randomized controlled studies are required to confirm these observations and determine the optimal injection level.

## Figures and Tables

**Table 1:** Demographic Data of the Groups.

Parameter	All Groups (*n* = 108)	Group I (*n* = 36)	Group II (*n* = 36)	Group III (*n* = 36)	*p*
Sex *n* (%)					0.933 ^¥^
Male	17 (15.7)	6 (16.7)	5 (13.9)	6 (16.7)	
Female	91 (84.3)	30 (83.3)	31 (86.1)	30 (83.3)	
Age					0.957 ^Σ^
Median (Min–Max)	64 (46–70)	65 (46–70)	64 (56–70)	64 (58–70)	
Mean ± SD	63.97 ± 4.10	63.89 ± 5.06	63.89 ± 3.91	64.14 ± 3.24	
Height (cm)					0.843 ^Σ^
Median (Min–Max)	162.5 (148–180)	163 (150–180)	162 (148–176)	162.5 (150–180)	
Mean ± SD	162.32 ± 6.59	162.67 ± 6.33	161.81 ± 6.62	162.50 ± 6.95	
Weight (kg)					0.972 ^Σ^
Median (Min–Max)	81 (58–110)	82.5 (63–100)	80 (58–110)	80 (63–110)	
Mean ± SD	82.13 ± 10.29	82.42 ± 8.26	81.83 ± 11.98	82.14 ± 10.57	
BMI (kg/m^2^)					0.431 ^Σ^
Median (Min–Max)	31 (22.83–45.78)	31.25 (22.83–40.13)	31.62 (24.45–45.78)	30.06 (25.76–42.2)	
Mean ± SD	31.55 ± 4.15	31.22 ± 3.30	32.28 ± 5.13	31.14 ± 3.83	
Duration of Surgery (min)					0.881 ^Σ^
Median (Min–Max)	122.5 (110–140)	122.5 (110–140)	120 (110–140)	125 (110–140)	
Mean ± SD	123.75 ± 8.38	124.17 ± 9.37	123.19 ± 7.85	123.89 ± 8.03	
ASA Score *n* (%)					0.819 ^¥^
ASA 2	90 (83.3)	29 (80.6)	30 (83.3)	31 (86.1)	
ASA 3	18 (16.7)	7 (19.4)	6 (16.7)	5 (13.9)	
Presence of Hypertension *n* (%)					0.355 ^¥^
No	27 (25)	10 (27.8)	11 (30.6)	6 (16.7)	
Yes	81 (75)	26 (72.2)	25 (69.4)	30 (83.3)	
Presence of Diabetes Mellitus *n* (%)					0.338 ^¥^
No	69 (63.9)	26 (72.2)	20 (55.6)	23 (63.9)	
Yes	39 (36.1)	10 (27.8)	16 (44.4)	13 (36.1)	
Presence of Coronary Artery Disease *n* (%)					0.309 ^¥^
No	92 (85.2)	28 (77.8)	32 (88.9)	32 (88.9)	
Yes	16 (14.8)	8 (22.2)	4 (11.1)	4 (11.1)	

*p* < 0.05 indicates a statistically significant difference. ^¥^: The statistical analysis was performed using the Pearson chi-square test. ^Σ^: The statistical analysis was performed using one-way analysis of variance (One-way ANOVA).

**Table 2:** Comparison of Postoperative VAS Scores among Groups at Different Time Points.

VAS Scores	All (*n* = 108)	Group I (*n* = 36)	Group II (*n* = 36)	Group III (*n* = 36)	*p* Value		
					1v2	2v3	1v3
1 h	7.31 ± 6.78	5.28 ± 5.60 ^a^	4.44 ± 5.04 ^a^	12.22 ± 6.81 ^b^	0.819	<0.001 *	<0.001 *
4 h	20.97 ± 11.33	16.81 ± 8.29 ^a^	13.33 ± 6.87 ^a^	32.78 ± 7.41 ^b^	0.129	<0.001 *	<0.001 *
8 h	30.37 ± 11.66	26.94 ± 8.72 ^a^	23.47 ± 7.64 ^a^	40.69 ± 10.50 ^b^	0.237	<0.001 *	<0.001 *
12 h	29.95 ± 11.14	27.22 ± 9.29 ^a^	24.31 ± 5.99 ^a^	38.33 ± 12.01 ^b^	0.391	<0.001 *	<0.001 *
24 h	19.40 ± 9.16	17.36 ± 7.70 ^a^	14.31 ± 6.88 ^a^	26.53 ± 8.18 ^b^	0.208	<0.001 *	<0.001 *

Values are presented as mean ± 95% confidence interval (CI). *: Indicates statistically significant *p*-values at the <0.05 level. Statistical analysis was performed using one-way analysis of variance (One-way ANOVA), followed by Tukey’s honestly significant difference (HSD) post hoc test. ^a,b^: Groups with different superscript letters indicate statistically significant differences at *p* < 0.05.

**Table 3:** Comparison of VAS Scores during Dynamic Movement Tests among Groups.

VAS Scores	All (*n* = 108)	Group I (*n* = 36)	Group II (*n* = 36)	Group III (*n* = 36)	*p* Value		
Dynamic Movement Tests					1v2	2v3	1v3
A1 Test	21.39 ± 9.52	19.44 ± 8.26 ^a^	16.39 ± 7.89 ^a^	28.33 ± 8.19 ^b^	0.251	<0.001 *	<0.001 *
A2 Test	27.08 ± 10.53	25.83 ± 8.82 ^a^	22.22 ± 8.66 ^a^	33.19 ± 11.03 ^b^	0.249	<0.001 *	<0.001 *
A3 Test	33.56 ± 11.13	31.53 ± 9.92 ^a^	27.78 ± 7.51 ^a^	41.39 ± 11.06 ^b^	0.227	<0.001 *	<0.001 *

Values are presented as mean ± 95% confidence interval (CI). *: Indicates statistically significant *p*-values at the <0.05 level. Statistical analysis was performed using one-way analysis of variance (One-way ANOVA), followed by Tukey’s honestly significant difference (HSD) post-hoc test. ^a,b^: Groups with different superscript letters indicate statistically significant differences at *p* < 0.05.

**Table 4:** Comparison of VAS Scores During Static Movement Tests among Groups.

VAS Scores	All (*n* = 108)	Group I (*n* = 36)	Group II (*n* = 36)	Group III (*n* = 36)	*p* Value		
Static Movement Tests					1v2	2v3	1v3
P1 Test	19.86 ± 9.03	18.33 ± 7.65 ^a^	14.72 ± 6.96 ^a^	26.53 ± 8.18 ^b^	0.114	<0.001 *	<0.001 *
P2 Test	20.60 ± 9.29	18.47 ± 7.25 ^a^	15.56 ± 6.84 ^a^	27.78 ± 8.98 ^b^	0.251	<0.001 *	<0.001 *
P3 Test	23.06 ± 9.99	21.25 ± 7.96 ^a^	17.36 ± 7.12 ^a^	30.56 ± 9.84 ^b^	0.125	<0.001 *	<0.001 *
P4 Test	28.24 ± 10.35	26.53 ± 9.55 ^a^	24.31 ± 7.09 ^a^	33.89 ± 11.60 ^b^	0.589	<0.001 *	<0.001 *

Values are presented as mean ± 95% confidence interval (CI). * Indicates a statistically significant difference at the <0.05 level. Statistical analysis was performed using one-way analysis of variance (One-way ANOVA), followed by Tukey’s honestly significant difference (HSD) post hoc test. ^a,b^ Groups with different superscript letters indicate statistically significant differences at *p* < 0.05. Pairwise comparisons are presented as: 1 = Group I, 2 = Group II, 3 = Group III.

**Table 5:** Comparison of Sensory Block Duration Time among Groups.

	All (*n* = 108)	Group I (*n* = 36)	Group II (*n* = 36)	Group III (*n* = 36)	*p* Value		
					1v2	1v3	2v3
Sensory Block Duration Time (hours)	4.45 ± 1.61	5.38 ± 1.33 ^a^	5.03 ± 1.29 ^a^	2.95 ± 0.95 ^b^	0.442	<0.001 *	<0.001 *

Values are presented as mean ± 95% confidence interval (CI). * Indicates statistically significant *p*-values at the <0.05 level. Statistical analysis was performed using one-way analysis of variance (One-way ANOVA), followed by Tukey’s honestly significant difference (HSD) post hoc test. ^a,b^: Groups with different superscript letters indicate statistically significant differences at *p* < 0.05. Pairwise comparisons are presented as: 1 = Group I, 2 = Group II, 3 = Group III.

**Table 6:** Comparison of Postoperative Tramadol Consumption among Groups.

	All (*n* = 108)	Group I (*n* = 36)	Group II (*n* = 36)	Group III (*n* = 36)	*p* Value		
					1v2	2v3	1v3
Patients Requiring Tramadol Hydrochloride, *n* (%)	94 (87)	30 (83.3)	28 (77.8)	36 (100)	--	--	--
Tramadol Hydrochloride Consumption	99.07 ± 58.79	80.56 ± 49.68 ^a^	75 ± 56.70 ^a^	141.67 ± 45.51 ^b^	0.888	<0.001 *	<0.001 *

Values are presented as mean ± 95% confidence interval (CI). * Indicates a statistically significant difference at the <0.05 level. Statistical analysis was performed using one-way analysis of variance (One-way ANOVA), followed by Tukey’s honestly significant difference (HSD) post hoc test. ^a,b^: Groups with different superscript letters indicate statistically significant differences at *p* < 0.05.

**Table 7:** Patients Requiring Tramadol According to Postoperative Time Intervals.

Time İnterval	Group I *n* (%)	Group II *n* (%)	Group III *n* (%)	*p* Value		
				1v2	1v3	2v3
0–4 h	1 (2.8) ^a^	1 (2.8) ^a^	6 (16.7) ^a^	1	0.108	0.108
4–8 h	5 (13.9) ^a^	3 (8.3) ^a^	18 (50.0) ^b^	0.710	0.002 *	<0.001 *
8–12 h	18 (50.0) ^a^	16 (44.4) ^a^	31 (86.1) ^b^	0.814	0.002 *	<0.001 *
12–24 h	10 (27.8) ^a^	8 (22.2) ^a^	22 (61.1) ^b^	0.786	0.005 *	<0.001 *

Values are presented as n (%). * Indicates statistically significant *p*-values at the <0.05 level. Statistical analysis was performed using Fisher’s exact test. ^a,b^: Groups with different superscript letters indicate statistically significant differences at *p* < 0.05. Pairwise comparisons are presented as: 1 = Group I, 2 = Group II, 3 = Group III.

## Data Availability

The data presented in this study are available on request from the corresponding author.

## Abbreviations

The following abbreviations are used in this manuscript:

TKA	Total Knee Arthroplasty
ACB	Adductor Canal Block
VAS	Visual Analog Scale
ERAS	Enhanced Recovery After Surgery
ASA	American Society of Anesthesiologists
SpO_2_	Peripheral Capillary Oxygen Saturation
mL	Milliliters
FA	Femoral Artery
SM	Sartorius Muscle
NSAIDs	Nonsteroidal Anti-inflammatory Drugs
TUG	Timed Up and Go
5 × SST	Five Times Sit-to-Stand Test
SLR	Straight Leg Raise
IBM	International Business Machines
SPSS	Statistical Package for the Social Sciences
ANOVA	Analysis of Variance
SD	Standard Deviation
BMI	Body Mass Index
MCID	Minimum Clinically Important Difference
CI	Confidence Interval
LAST	Local Anesthetic Systemic Toxicity
HSD	Honestly Significant Difference
NRS	Numeric Rating Scale
